# A Genomic Tool to Tackle Cryptic Diversity Demonstrates the Potential for Off‐Target Use of GT‐Seq Panels

**DOI:** 10.1111/1755-0998.70203

**Published:** 2026-09-24

**Authors:** Amanda S. Ackiss, Mark R. Vinson, Ann J. Ropp, Kristen M. Gruenthal, Trevor J. Krabbenhoft, Joseph V. Siegel, Wendylee Stott, Daniel L. Yule, Wesley A. Larson

**Affiliations:** ^1^ U.S. Geological Survey Great Lakes Science Center Ann Arbor Michigan USA; ^2^ Wisconsin Cooperative Fishery Research Unit, College of Natural Resources University of Wisconsin‐Stevens Point Stevens Point Wisconsin USA; ^3^ U.S. Geological Survey Great Lakes Science Center, Lake Superior Biological Station Ashland Wisconsin USA; ^4^ Alaska Department of Fish and Game, Division of Commercial Fisheries Gene Conservation Laboratory Juneau Alaska USA; ^5^ Wisconsin Department of Natural Resources, Office of Applied Science College of Natural Resources, University of Wisconsin‐Stevens Point Stevens Point Wisconsin USA; ^6^ Department of Biological Sciences University at Buffalo Buffalo New York USA; ^7^ Biological Sciences Department SUNY Oswego Oswego New York USA; ^8^ Fisheries & Oceans Canada Pêches et Océans Canada Winnipeg Manitoba Canada; ^9^ National Oceanographic and Atmospheric Administration National Marine Fisheries Service, Alaska Fisheries Science Center, Auke Bay Laboratories Juneau Alaska USA

**Keywords:** amplicon sequencing, cross‐amplification, genetic stock identification, species complex

## Abstract

A comprehensive understanding of life history is vital to successful species conservation and management. When different life history stages are accompanied by considerable morphological or cryptic variation, such as the egg and larval phases exhibited by most fishes, genomic tools are essential for identifying species so that early‐life ecology questions can be studied. Genotyping‐in‐thousands by sequencing (GT‐seq) has recently emerged as a targeted and efficient approach for species identification. We leveraged existing genomic and transcriptomic data to develop a GT‐seq panel capable of differentiating the members of the *Coregonus artedi* complex, a radiation of salmonids in the Laurentian Great Lakes whose members are indistinguishable with mitochondrial DNA barcoding loci and are the focus of bi‐national conservation initiatives. Our panel of 494 loci was able to assign fishes in the *C. artedi* complex to species and lake. We examined cross‐amplification in other coregonines with overlapping distributions and found that congeneric Lake Whitefish ( *C. clupeaformis* ) cross‐amplified at 94% of loci and confamilial Round and Pygmy Whitefish (*Prosopium* spp.) cross‐amplified at 42% and 38% of loci, respectively. We adapted bioinformatic probes to account for *Prosopium*‐specific variants including 22 new SNPs and developed a whitelist of 428 SNPs capable of distinguishing these whitefishes. Finally, we demonstrated performance by identifying 3066 coregonine larvae and juveniles collected in spring 2019–2021 from Lake Superior. These results hold promise for future insights into the species‐specific ecology of early life coregonines and demonstrate the flexibility of GT‐seq panels, which may cross‐amplify hundreds of informative genome‐wide loci in related taxa.

## 1 Introduction

Cryptic diversity poses many challenges to successful species conservation and management (Hending 2025). The ability to identify species throughout their life cycle is vital to understanding their ecology and biology, but if a species is elusive (Claridge et al. 2004) or its phenotypes are visually indistinguishable (Bickford et al. 2007), fundamental gaps in knowledge can arise with potentially dire consequences (Geller 1999; Schönrogge et al. 2002; Nguyen et al. 2019). In many species, cryptic diversity may be especially prevalent in early life stages such as propagules and larvae (Pfenninger et al. 2007; Collin et al. 2020; Hoffman et al. 2021). In others, cryptic diversity occurs in adults where morphological differences may be negligible (Hebert et al. 2004; Vega‐Sánchez et al. 2024). In both cases, molecular markers have become one of the most reliable means for detecting cryptic diversity (Hebert et al. 2003; Stoeckle 2003; Bickford et al. 2007), and high‐throughput methods for species identification at both individual and community levels are becoming increasingly common with decreasing sequencing costs (Kimmerling et al. 2018; Porter and Hajibabaei 2018; Miya et al. 2020; Hebert et al. 2025).

Recent evolutionary divergence has been identified as a potential mechanism causing cryptic diversity (Chenuil et al. 2018; Struck et al. 2018; Cahill et al. 2024), and it can complicate genetic species identification even in the absence of morphological ambiguity. Success rates for genetic identification of recently diverged species with standard mitochondrial, chloroplast and nuclear DNA barcoding markers have been shown to be significantly lower than for evolutionarily older species due to factors such as incomplete lineage sorting (van Velzen et al. 2012). In such cases, increasing marker density across the genome improves discrimination of distinct evolutionary units. For example, two of the four subspecies in the North American yellow‐rumped warbler ( *Setophaga coronata* ) species complex could not be reliably distinguished with genetic methods until high throughput, reduced‐representation or whole genome sequencing was used (Toews et al. 2016; Szarmach et al. 2021). Similarly, restriction site‐associated DNA sequencing (RAD‐seq) was able to completely resolve reciprocal monophyly across 16 recently diverged Lake Victoria cichlids for the first time (Wagner et al. 2013).

Reduced representation and whole genome sequencing are powerful methods to address the challenges of characterizing cryptic or recently diverged populations and species and are more accessible than ever for non‐model organisms (Ekblom and Galindo 2011; Ellegren 2014); however, more cost‐effective approaches for genetic identification are needed when repeated or large numbers of samples are required to address specific questions. Over the last decade, targeted amplicon panels such as genotyping‐in‐thousands by sequencing (GT‐seq; Campbell et al. 2015) have emerged as a practical approach to combining the power of genome‐wide loci and the need for fast, cost‐effective genotyping (Meek and Larson 2019). Developed from reduced representation sequencing (Bootsma et al. 2020; Euclide et al. 2022; Hayward et al. 2022; Homola et al. 2025) and whole genome sequencing (Beemelmanns et al. 2025) datasets, amplicon panels can be designed to target both neutral and adaptive loci (Garrett et al. 2024; Schmidt et al. 2020) enabling a broad range of population and demographic inference. Since the amplification target is a sequence rather than specific single nucleotide polymorphisms (SNPs), which are the target of widely used, cost‐effective assays such as TaqMan and high‐density SNP chips (Francisco et al. 2005; Gunderson et al. 2006; Groenen et al. 2011), amplicons that contain multiple SNPs confer the additional benefit of calling microhaplotypes (Baetscher et al. 2018, 2023). As a growing number of amplicon panels are developed for genetic monitoring, there is potential for leveraging existing panels for off‐target species through cross‐amplification as was commonly done during the age of microsatellites (Moore et al. 1991; Schlotteröer et al. 1991; Primmer et al. 1996; Wilson et al. 2004).

The *Coregonus artedi* species complex, a radiation of salmonids in the Laurentian Great Lakes of North America known as ciscoes, embodies the challenges of both cryptic diversity and recent evolutionary divergence. The Coregoninae subfamily underwent repeated diversification events in cold‐water habitats across the northern hemisphere after the Last Glacial Maximum (Hudson et al. 2007), and the short evolutionary timescale across which this diversification occurred has led to uninformative and often conflicting results when using classical genetic and morphological data to infer species relationships (Nikolsky and Reshetnikov 1970; Reed et al. 1998; Turgeon et al. 1999; Turgeon and Bernatchez 2003), a situation referred to as the ‘coregonid problem’ (Svärdson 1949; Stott and Todd 2007; Mee et al. 2015). Additionally, in the Great Lakes, many deepwater species in this complex can be difficult to visually distinguish (Koelz 1929), and there are no morphological characters that can reliably identify the eggs or larvae (Auer 1982; George et al. 2018), rendering critical gaps in our understanding of species‐specific early life history. Recent studies on Great Lakes ciscoes employing RAD‐seq (Ackiss et al. 2020; Lachance et al. 2021), RNA‐seq (Bernal et al. 2022), and whole genome sequencing (Backenstose et al. 2024) have enabled reliable and accurate species identification, particularly among deepwater species, for the first time.

A century of severe declines in the diversity of Great Lakes ciscoes (Eshenroder et al. 2016) has led to the development of a bi‐national framework to support coregonine restoration (Bunnell et al. 2023). The framework recognizes a need to resolve species relationships with genetics and augments previously identified research needs for improving our understanding of coregonine ecology, including species‐specific larval emergence timing, dispersal and duration and the influence of environmental and invasive species interactions on recruitment variability (Zimmerman and Krueger 2009). We leveraged recent genomic and transcriptomic datasets to develop a GT‐seq panel capable of discriminating ciscoes that will allow scientists to efficiently address these questions at a basin‐wide scale. Since early life stages (larvae, eggs) could be confused with those of sister species, Lake Whitefish ( *C. clupeaformis* ), Round Whitefish ( *Prosopium cylindraceum* ) and Pygmy Whitefish ( *P. coulterii* ), we also tested the cross‐amplification of panel loci and successfully modified a subset of genotyping probes for whitefish species identification. Finally, we used the panel to identify 3066 larval and age‐1 coregonines sampled in Lake Superior in 2019–2021, enabling the first large scale examination of early life diversity and distribution in Great Lakes ciscoes. Our results lay the foundation for a myriad of insights into coregonine early life history that can inform their conservation and restoration, and we demonstrate both the value of flexible, cost‐effective genetic tools for elucidating whole‐life history when cryptic early life stages are present as well as the ease with which GT‐seq panels can be adapted for off‐target use.

## 2 Methods

### 2.1 Development of an Amplicon Panel From RAD‐Seq Data

**FIGURE 1:**
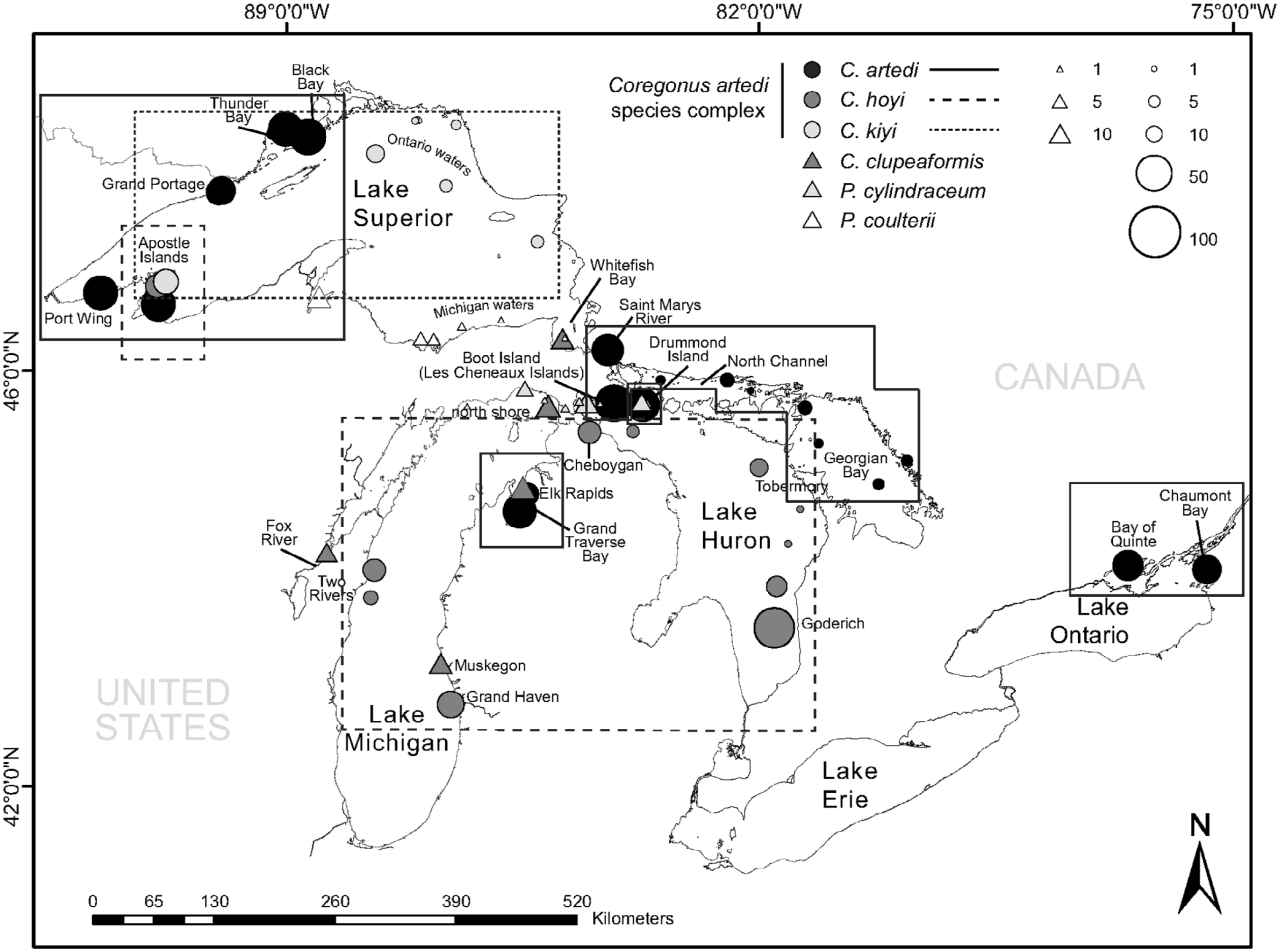
Map of collection sites across the Great Lakes for samples used in GT‐seq panel development (circles) and cross‐amplification testing (triangles). Boxes indicate reporting groups for Cisco *C. artedi* (solid lines), Bloater *C. hoyi* (dashed lines), and Kiyi *C. kiyi* (dotted line). Sizes of collection site markers scaled to number of samples (see Table 1 for collection and reporting group details).

Candidate loci for an amplicon panel for genetic stock identification (GSI) of Great Lakes ciscoes were identified from RAD‐seq data from sampled adults representing the three major extant species in the *Coregonus artedi* complex ( *C. artedi* , *C. hoyi* , *C. kiyi* ). Two other species, *C. zenithicus* and *C. nigripinnis* , have uncertain status in Lake Superior and may potentially be extinct (Eshenroder et al. 2016). Fishes field identified as *C. zenithicus* and *C. nigripinnis* are caught extremely rarely, and RAD‐seq data from these fishes lend uncertainty as to whether those field identifications are correct (Ackiss et al. 2020), therefore, *C. zenithicus* and *C. nigripinnis* were not included in this analysis. Lake‐specific representatives of each species were also included (Superior, Michigan, Huron and Ontario, Figure 1; Table 1). DNA from samples was extracted largely from fin clips (or rarely from heart, liver, or muscle tissue) using Qiagen DNeasy Blood & Tissue kits, quantified using a Quant‐it PicoGreen dsDNA Assay (Invitrogen; Waltham, MA), and normalized to 200 ng for bestRAD library preparation which followed the same protocols outlined by Ackiss et al. (2020). Libraries were sequenced through Novogene (Sacramento, CA) using paired‐end (PE) 150 technology on the Illumina Novaseq 6000 platform.

**TABLE 1:** Collections used for GT‐seq panel development (identification and simulated performance of candidate loci from RAD‐seq data) and cross‐amplification testing on sister species and genera. Lake codes are Huron (H), Ontario (O), Michigan (M), Superior (S), N is the number of genotyped samples from each population, na = not applicable.

Panel stage	Reporting unit	Species	Lake	Sample location	Collection date	N
Development	A‐HBI‐SM‐NC	*C. artedi*	H	Boot Island	Nov 2015	56
				Saint Marys River	Nov 2010	40
				Georgian Bay	Jul 2017–18, Sep 2017	26
				North Channel	Aug, Sep 2017	14
	A‐HDI	*C. artedi*	H	Drummond Island	Oct 2010	42
	A‐MCH	*C. artedi*	M	Elk Rapids	Nov 2015–17	21
				Grand Traverse Bay	Dec 2013	46
	A‐ONT	*C. artedi*	O	Chaumont Bay	Dec 2016	35
				Bay of Quinte	Nov 2013	39
	A‐SUP	*C. artedi*	S	Apostle Islands	Nov 2017	60
				Black Bay	Nov 2010	52
				Grand Portage	Oct 2017	58
				Port Wing	Nov 2010	47
				Thunder Bay	Nov 2017	66
	H‐HUR‐MCH	*C. hoyi*	H	Cheboygan	Sep 2014, 2016	25
				Goderich	Oct 1998	64
				Tobermory	Sep 2016	33
			M	Grand Haven	Aug 2014	28
				Two Rivers	Feb 2018	27
	H‐SST	*C. hoyi*	S	Apostle Islands	July 2005	21
	K‐SUP	*C. kiyi*	S	Apostle Islands	July 2005	25
				Ontario waters	Sep 2011	31
Cross‐amplification testing	na	*C. clupeaformis*	M	Fox River	Nov 2017	23
				Muskegon	Oct 2018	13
				Elk Rapids	Nov 2018	18
				North shore	Dec 2019	17
	na	*C. clupeaformis*	S	Whitefish Bay	Nov 2008	14
	na	*P. cylindraceum*	H	Les Cheneaux Islands region	Jul, Aug, Sep 2021	26
			M	North Shore	Jul, Aug 2021	10
	na	*P. coulterii*	S	Michigan waters	Jun 2021	13
			S	Sand Bay	Jun 2023	12

Reads were processed in STACKS to identify variant loci using the same parameters for the subprograms *process_radtags*, *ustacks*, *cstacks*, *gstacks* and *populations* described in Ackiss et al. (2020). The *de novo* catalogue generated in *cstacks* was created with five individuals from each unique species and lake. Iterative SNP filtering was performed with vcftools v0.1.15 (Danecek et al. 2011) and included (1) removing loci with a minor allele count less than three, (2) removing loci genotyped in fewer than 30% of individuals, (3) removing individuals missing more than 50% of loci and (4) removing remaining loci genotyped in fewer than 70% of individuals. Putatively paralogous loci were identified with the program HDPlot (McKinney et al. 2017) and filtered using cutoffs of heterozygosity > 0.55 and a read ratio deviation > 5 and < −5. In order to identify candidate loci for genetic stock identification (GSI) and the reporting groups to test them, G‐statistics (analogues of F‐statistics based on Nei 1987) including differentiation (*G'*_ST_), inbreeding coefficients (*G*_IS_) and observed and expected heterozygosity (*H*_o_/*H*_e_), were calculated for each locus and collection in Genodive v3.0 (Meirmans 2020).

### 2.2 Simulated Performance of Candidate Panels for Genetic Stock Identification

Reporting groups for assessing candidate panel performance were created by grouping individuals from unique collection sites and species with pairwise *G'*_ST_ > 0.015. Self‐assignment of individuals to reporting groups was assessed using a leave‐one‐out procedure with the *self_assign* function in the R package *rubias* (Moran and Anderson 2019). Candidate panels for genetic stock identification of 600 RAD‐seq loci each were designed with different combinations of high *G'*_ST_ loci and high diversity loci generated from basin‐wide and Lake Superior only datasets as both SNP and haplotype test datasets (File S1, Table S1), following similar protocols outlined by Bootsma et al. (2020) and Euclide et al. (2022). The performance of candidate panels was simulated using the *assess_reference_loo* function in *rubias* (mix size = 200, MCMC simulations = 1000).

### 2.3 GT‐Seq Panel Development

Primers for candidate loci were designed in Geneious Prime v.2019.2 using locus consensus sequences pulled from the STACKS catalogue (catalogue.fa) and a vcf file containing all polymorphic sites on a target locus. Primer design parameters included an optimal product size of 100 bp (min 50 bp, max 150 bp) and optimal probes with a size of 20 bp (min 18 bp, max 25 bp), Tm of 60°C (min 57°C, max 63°C), GC content of 50% (min 40%, max 60%), and homopolymers (poly‐X) no longer than three bp. The Test Primers tool in Geneious was used to check whether primers would bind non‐target loci in the pool of consensus sequences. Primers binding non‐target fragments were redesigned, and if redesign did not fix non‐target binding or was not possible, the locus was dropped. Primer design occurred iteratively; as loci were dropped due to lack of optimal priming sites or primers that bound non‐target fragments, the locus with the next highest *G'*_ST_ value was selected for primer design until a total of 600 primer sets was reached. In addition to RAD‐seq loci, primers were also designed for 45 loci from transcriptomic data that contained a polymorphic site exhibiting high differentiation among *C. artedi* , *C. hoyi* and *C. kiyi* in Lake Superior (Bernal et al. 2022). Primer design for transcriptomic data followed the same protocol as above, using cDNA sequences annotated with a vcf file containing information on target variant location.

GT‐seq panel optimization was performed following the methods described by Bootsma et al. (2020) modified from the original methods of Campbell et al. (2015). Briefly, samples were amplified at panel loci, barcoded with i5/i7 indices, normalized and pooled into master libraries for sequencing with PE 150 chemistry on the Illumina sequencing platform. Detailed panel amplification parameters can be found in Supporting Information (File S2). Locus genotyping and evaluation were performed using the GTscore pipeline (https://github.com/gjmckinney/GTscore), which uses a Perl script and probe file designed to quickly identify loci and variants from a list of forward primer sequences and 20 bp probe sequences that identify target alleles. The original 645 GT‐seq panel loci were iteratively reduced over two rounds of test amplification. Over‐represented loci were identified and removed after each round of test amplification and sequencing. The STACKS catalogue sequences for the final subset of panel loci were mapped to the *C. artedi* genome (GCA_039881085.1; Backenstose et al. 2024) with BWA fastmap (Li 2013) to characterize coverage across the genome. Loci composed of super‐maximal exact matches with 2 or fewer mismatches to chromosomes were visualized with karyoploteR (Gel and Serra 2017).

### 2.4 Genotyping Reference Individuals and Testing Cross‐Amplification

We selected a subset of ciscoes to serve as reference individuals for genetic assignments and genotyped them with the final panel of GT‐seq loci. Reference individuals were comprised of adult fishes not used in the RADseq analysis for panel development that were morphologically identified as Cisco *C. artedi* (*n* = 30), Bloater *C. hoyi* (*n* = 49) and Kiyi *C. kiyi* (*n* = 50) and collected from Lake Superior between 2014 and 2021 (ScienceBase, https://doi.org/10.5066/P13W68YB). DNA extraction of tissue from reference individuals was performed with Qiagen DNeasy Blood & Tissue kits following the manufacturer's protocols with a final elution in 200 ul Tris low‐EDTA (TLE, 10 mM Tris–HCl, 0.1 mM EDTA) buffer.

To explore cross‐amplification of panel loci in other coregonines that can be difficult to distinguish from ciscoes in early life stages, we tested panel amplification on the other three coregonines present in the Great Lakes: Lake Whitefish ( *Coregonus clupeaformis* ), Round Whitefish ( *Prosopium cylindraceum* ) and Pygmy Whitefish ( *P. coulterii* ). Lake Whitefish samples included adults collected from four locations in Lake Michigan between 2017 and 2019 (*n* = 72) and one location in Lake Superior in 2008 (*n* = 14). *P. cylindraceum* samples included adults collected in Lake Michigan and Lake Huron in 2021 (*n* = 36). *P. coulterii* , which are only present in Lake Superior, were also collected in 2021 and 2023 (*n* = 25). Collection locations of outgroup samples are shown in Figure 1, and sample metadata are archived on ScienceBase (https://doi.org/10.5066/P13W68YB). DNA extraction of outgroup sample tissues was performed with Qiagen DNeasy Blood & Tissue kits as described for reference ciscoes, except for the Lake Whitefish samples from Lake Michigan, which were extracted using a 10% Chelex 100 (200–400 mesh; Biorad, Hercules, CA) slurry as described in Bootsma et al. (2020).

GT‐seq libraries for all reference individuals were prepared with the final panel following the protocol described in File S2. Master library pools were PE150 sequenced with v2 chemistry on an Illumina MiSeq at the US Geological Survey's Great Lakes Science Center (Ann Arbor, Michigan, USA). Genotypes were called from forward reads using a probe file in GTscore.

### 2.5 Quantifying Cross‐Amplification Success and Modifying Probes for Outgroup Assignment

We quantified the success of cross amplification of the GT‐seq panel on congener and confamilial whitefishes with three methods. First, we evaluated the number of loci and SNPs that were genotyped for Great Lakes whitefishes in GTscore with a file used to call SNPs via 20 bp in silico probes for identifying ciscoes. Each whitefish species was run separately, and loci and SNPs that genotyped in > 50% of samples were counted. GT‐seq loci that cross‐amplified were annotated on the 38 chromosomes of the *C. artedi* genome with karyoploteR (Gel and Serra 2017). We mapped outgroup reads to the FASTA catalogue of loci in the panel with BWA mem (Li 2013) and examined loci that cross‐amplified in whitefishes in IGV. Our intent was to modify existing panel probes to expand their capacity for whitefish identification rather than to create entirely new probes which could introduce variability to our panel and potentially reduce accuracy for identification of ciscoes. If existing SNP probes overlapped a variant that was present in a whitefish species, the probe was modified to account for and/or call the SNP. Probes that overlapped multiple whitefish SNPs were not redesigned. The newly modified probe file was used to genotype references with GTscore, and genotyping success was examined in the *dartR* package in R (Gruber et al. 2018; Mijangos et al. 2022). The number of panel loci that cross‐amplified in Round and Pygmy Whitefishes (*Prosopium* spp.) was much lower than the number that cross‐amplified in sister species Lake Whitefish (Coregonus); therefore, we tested the genetic assignment power of (1) a whitelist of the panel loci that were polymorphic in a dataset comprised of all whitefishes and (2) a whitelist of the panel loci that were polymorphic in a dataset comprised of only *Prosopium* whitefishes. For both, individuals that were missing > 25% of the data were dropped and assignment accuracy was tested with the assign.MC function in the R package *assignPOP* (Chen et al. 2018). Training data included proportions of 0.5, 0.7 and 0.9 for individuals and 0.1, 0.25, 0.5 and 1 for loci over 30 iterations with the support vector machine (svm) predictive model.

Second, we mapped forward reads from Great Lakes whitefishes to the *C. artedi* genome to quantify locus amplification and number of variant sites sampled within species. Adaptor sequence was trimmed from raw reads with cutadapt v5.0 (Martin 2011), and reads were quality filtered with fastp v0.23.4 (Chen 2023; parameters: −5 20, −3 20, ‐cut_window_size 5, ‐cut_mean_quality 15, −q 15). The forward read fastq files were mapped to the genome with BWA mem (Li 2013). Variant calling was performed with Freebayes v1.3.6 (Garrison and Marth 2012) on merged bam files for each species and data were exported as VCF files. Parameters used include a minimum base quality of 20 (−q), minimum mapping quality of 20 (−m), minimum coverage of 20 (‐min‐coverage), variants with 3 bp called as a single contiguous haplotype (−E), and read placement/strand bias ignored during calculation of SNP site quality scores (‐V). VCFtools (Danecek et al. 2011) was used to reduce variants to bi‐allelic SNPs, followed in order by (1) filtering SNPs with a minor allele count of at least 3, (2) dropping individuals that were genotyped in fewer than 50% of loci, (3) dropping loci genotyped in fewer than 80% of remaining individuals and (4) dropping loci with a minor allele frequency of less than 0.01.

Finally, to examine potential for cross amplification in non‐Great Lakes *Coregonus* spp., the final subset of panel amplicons were mapped to the European Whitefish *C. lavaretus* sp. ‘Balchen’ genome (GCA_902810595.1, De‐Kayne et al. 2020), the Amur Whitefish *C. ussuriensis* genome (GCA_031479575.1, Huang et al. 2024), and the Lake Whitefish *C. clupeaformis* genome (GCA_018398675.1, Mérot et al. 2023) with BWA mem (Li 2013). Mapping success of European and Amur whitefishes was compared to mapping success in Lake Whitefish which could be benchmarked with genotyping rates.

### 2.6 Collection, Processing and GT‐Seq Library Preparation of Larval and Age‐1 Coregonines in Lake Superior

Collection of larval and age‐1 ciscoes in 2019 and 2021 and age‐1 ciscoes in 2020 was conducted by the U.S. Geological Survey, Great Lakes Science Center during annual surveys of Lake Superior. Larvae were collected with a paired 1 m^2^, 500 μm mesh neuston net (SEA‐GEAR Corporation). The bottom of the net frame was fished ~0.5 m below the lake surface. The net was fished for 10 min at ~4.0 km per h for ~0.7 km as determined from the global positioning system on the research vessel. Age‐1 ciscoes were collected by bottom trawling using a 12 m‐wide Yankee bottom trawl (Vinson et al. 2021). Total lengths (TL, mm) of 2019 larvae were measured in the laboratory. Larvae that could fit within the microscope field of view at the broadest magnification (8×) for the Leica EZ4W were photographed, and TL was measured to the nearest 0.01 mm with calibrated distance lines in Leica Application Suite v3.4.0 (Leica Microsystems; Switzerland). Larvae whose size exceeded the field of view were measured with digital callipers. TL of 2021 larvae was measured to the nearest mm using a standard ruler under a dissecting microscope. TL measurements for 2019–2021 age‐1 coregonines were made at the time of collection using a standard ruler at the time of capture.

Preliminary funding was limited for processing 2019 samples, so tissue from up to 12 randomly selected 2019 larval coregonines from each neuston net tow (*n* = 912) was subsampled for GT‐seq genotyping. All collected 2019–2021 age‐1 coregonines (*n* = 360), and all collected 2021 larval coregonines (*n* = 1872) were processed. DNA from larval and age‐1 samples was extracted using either a 10% Chelex 100 slurry or Qiagen DNeasy Blood & Tissue kits as described for reference and outgroup coregonines above. Libraries for larval and age‐1 samples were prepared following the same protocol described above for panel optimization (File S2). The libraries for 2019 larval and age‐1 samples were PE 150 sequenced with v1 chemistry on an Illumina NovaSeq platform at the UW Biotechnology Center DNA Sequencing Core Facility (Madison, Wisconsin, USA). The libraries for 2020 and 2021 samples were PE150 sequenced with v2 chemistry on an Illumina MiSeq at the US Geological Survey's Great Lakes Science Center (Ann Arbor, Michigan, USA).

### 2.7 GT‐Seq Genotyping and Genetic Identification of Early Life Coregonines in Lake Superior

Raw sequencing data for references and larval and age‐1 coregonines were processed and genotyped in GTscore. Samples with outlying heterozygosity and/or a contamination score, which is the proportion of heterozygous genotypes whose allele ratios significantly differ from 1:1 (https://github.com/gjmckinney/GTscore), of > 0.50 were dropped before converting genotypes to genepop format. Genotypes were imported into *dartR* (Gruber et al. 2018; Mijangos et al. 2022). First, to determine if Lake, Round, or Pygmy Whitefishes were present in our samples, we reduced the dataset to the final whitefish whitelist and kept individuals genotyped in at least 25% of the loci. We exported reference and unknown data in genepop format and used *assignPOP* (Chen et al. 2018) to identify whitefishes. *C. artedi* , *C. hoyi* and *C. kiyi* references were combined into a single reference population for this analysis. The *assign.X* function was run using the default criterion for retaining the number of principal components (‘Kaiser‐Guttman’) and the support vector machine (svm) predictive model. Whitefishes with an assignment probability > 70% to a reference group were assigned to that group.

Identified whitefishes were removed from the full dataset, and iterative filtering was repeated in *dartR* to generate a dataset for genetic assignment of ciscoes. Filtering steps included removing loci missing in more than 25% of individuals, followed by removing individuals missing more than 62.5% of genotypes. Finally, loci genotyped in less than 75% of the remaining individuals were dropped to generate the final dataset. Genetic similarity of genotyped individuals was examined with principal components analysis (PCA) using the glPCA function before performing genetic assignments.

Preliminary genetic assignments to adult *C. artedi* , *C. hoyi* and *C. kiyi* references were performed with *assignPOP* (Chen et al. 2018) following the same methods as described for whitefish identification. Since hybridization among ciscoes has been documented (Ackiss et al. 2020; Lachance et al. 2021), final genetic assignments were generated with model‐based clustering without prior population information in STRUCTURE v2.3.4 (Pritchard et al. 2000; Falush et al. 2003, 2007) to infer ancestry proportions and estimate admixture. Given the large number of assignments to be performed, our dataset was randomly subsampled to batches of ~100 unknowns to be run with the reference individuals (*n* = 129). STRUCTURE was run with the admixture model and correlated allele frequencies using a burn‐in of 50,000 followed by 100,000 MCMC steps. Cluster analysis on each batch was run for K = 3 with 5 replicates. Replicates were merged with the R package *pophelper* (Francis 2017), and larvae and juveniles with Q‐scores > 70% to a reference group cluster were assigned to that cluster following Ackiss et al. (2020) and Lachance et al. (2021). Individuals that did not assign with high probability to a single cluster were labelled as putative hybrids.

### 2.8 Preliminary Analysis of Early Life Coregonines in Lake Superior

Identified larvae from 2019 to 2021 were plotted to show the composition of species at each collection location in Lake Superior. The spatial (whole lake) and temporal (May 29–July 23) spread of larval collections was broadest in 2019 because the extent of these surveys was not impacted by the restrictions introduced during the COVID pandemic in 2021 collections. Therefore, we used the 2019 cohort data to examine changes in total length (TL) over time. We extrapolated larval growth rate per day (mm/day) using linear regression for the first month of collections, which is more likely to represent a demographic cohort. Identified age‐1 s from 2019 to 2021 were also plotted to show the composition of species at each collection location, and genetic identifications were used to assess the accuracy of field identifications in 2019–2020 age‐1 s.

## 3 Results

### 3.1 Development of an Amplicon Panel From RAD‐Seq Data

A total of 858 samples from the three major extant species in the *Coregonus artedi* species complex representing all the lakes with existing populations (Figure 1) were genotyped at 49,841 SNPs using RAD‐seq. Ten initial reporting groups were identified with a threshold of *G*_ST_ ≥ 0.15 (File S1, Figure S1). Results indicated a large proportion of individuals from Grand Portage in Lake Superior (A_SGP, 33%) and the North Channel and Georgian Bay regions in Lake Huron (A_HNC, 25%) assigned to nearby clusters—potentially because these collections were comprised of non‐spawning period fish. These locations were combined with their counterparts to generate eight final reporting groups (File S1, Figure S1B Table 1), including one for each species in Lake Superior, one for *C. hoyi* from Lake Michigan and Lake Huron, one for Lake Michigan *C. artedi* , two for Lake Huron *C. artedi* and one for Lake Ontario *C. artedi* (Figure 1). The results from candidate panels indicated similar performance between a panel comprised of 500 high *G'*_ST_ SNPs among Lake Superior ciscoes and 100 SNPs with high basin‐wide *H*_e_ and a panel comprised of both 500 high *G'*_ST_ SNPs and 100 SNPs with high *H*_e_ from Lake Superior ciscoes (File S1, Table S1, Figure S2). We selected the latter for designing primers along with the 45 high *F*_ST_ transcriptomic loci.

**FIGURE 2:**
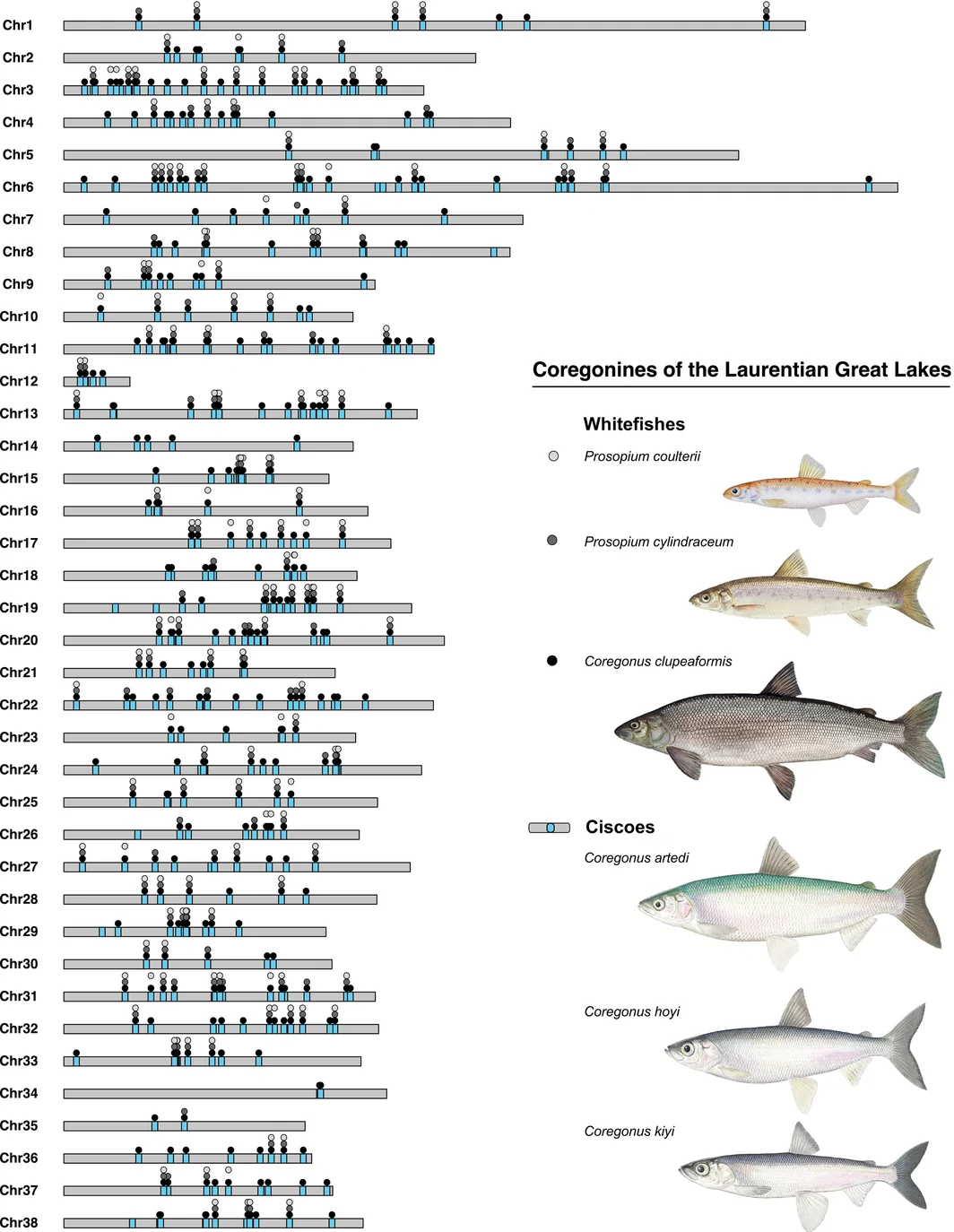
Cross amplification in Great Lakes coregonines and location of the GT‐seq panel loci in the *C. artedi* genome. Loci that cross‐amplified in whitefishes are annotated with circles—Pygmy Whitefish *P. coulterii* (light grey), Round Whitefish *P. cylindraceum* (dark grey), Lake Whitefish *C. clupeaformis* (black).

The original 645 GT‐seq panel loci were iteratively reduced over three rounds of test amplification. Over‐represented loci were identified and removed after each round of test amplification, resulting in a final panel of 494 loci composed of 467 RAD‐seq based loci and 27 RNA‐seq based loci containing 1220 target SNPs. A total of 434 panel loci (87.5%) mapped with 2 or fewer mismatches to the *C. artedi* genome across all 38 chromosomes (Figure 2).

### 3.2 Quantifying Cross‐Amplification Success and Modifying Probes for Outgroup Assignment

After reviewing cross‐amplifying loci, existing panel probes were modified to account for 22 additional whitefish variants bringing the total number of target SNPs to 1242 (Files S1, S3, S4, Figure S3). Lake Whitefish *C. clupeaformis* genotyped at 464 panel loci (93.9%) at 1101 of the target SNPs with an average genotype rate of 93.9% (range 0.51–100%). Round Whitefish *P. cylindraceum* genotyped at 205 (41.5%) of panel loci at 404 of the target SNPs with an average genotype rate at these loci of 85.3% (range 0.50%–97.4%). Pygmy Whitefish *P. coulterii* genotyped at 186 (37.7%) of panel loci at 338 of the target SNPs with an average genotype rate at these loci of 75.9% (range 0.50%–90.6%). Cross‐amplifying loci in Lake Whitefish mapped to all 38 chromosomes of the *C. artedi* genome. Cross‐amplifying loci in Round and Pygmy Whitefish mapped to 36 and 35 chromosomes, respectively. Locations of cross‐amplifying loci in whitefishes are annotated in Figure 2.

A whitelist of 571 polymorphic SNPs across all whitefishes and a whitelist of 428 polymorphic SNPs in *Prosopium* whitefishes performed with 100% assignment accuracy for Lake Whitefish, Round Whitefish and Pygmy Whitefish at all combinations of training data and loci. Since *Prosopium* whitefishes generally cross‐amplify in fewer loci, we proceeded with the 428 SNP *Prosopium* whitefish‐specific whitelist (File S5) in downstream analyses to reduce the potential for losing samples through the missing data (> 25% loci) filter. The assignment accuracy plot and a PCA of reference individuals using the 428 SNP whitelist is reported in File S1 (Figures S4 and S5).

When sequenced amplicons were mapped to the *C. artedi* genome and variants were called and filtered within species, Lake Whitefish produced a dataset of 1280 bi‐allelic SNPs with a genotype rate of > 80% across 56 individuals. Round Whitefish and Pygmy Whitefish produced datasets of 124 and 297 bi‐allelic SNPs with a genotype rate of > 80% across 34 and 22 individuals, respectively. A summary of GT‐seq panel cross‐amplification in related Great Lakes coregonines is presented in Table 2.

**TABLE 2:** Cross‐amplification of the GT‐seq panel on other Great Lakes coregonines. Summary includes the number (percent) of the 494 panel loci that cross‐amplified, the number (percent) of the genotyped SNPs called from GT‐seq panel probes, the number of chromosomes of the *C. artedi* genome cross‐amplifying loci were found in (number of chromosomes to which panel amplicons mapped in the species' respective genome), and the number of biallelic SNPs within species after filtering when amplicons were mapped to the *C. artedi* genome with the number (*n*) of genotyped individuals.

Species	Common name	GT‐seq panel			Genome mapped
		Amplified loci	Genotyped SNPs	Chrom	Filtered SNPs (*n*)
*C. clupeaformis*	Lake Whitefish	464 (93.9%)	1101 (90.2%)	38 (39)	1280 (56)
*P. cylindraceum*	Round Whitefish	205 (41.5%)	404 (33.1%)	36 (−)	124 (34)
*P. coulterii*	Pygmy Whitefish	186 (37.7%)	338 (27.7%)	35 (−)	297 (22)

Finally, mapping panel amplicons to whitefish genomes suggests cross‐amplifying loci are widespread across genomes. When amplicons were mapped to the European Whitefish *C. lavaretus* sp. ‘Balchen’ genome, 443 panel loci (89.7%) had a mapping quality (MAPQ) score of ≥ 30 to regions in 39 out of 40 chromosomes. No matches occurred in chromosome 40, the smallest chromosome in the *C. lavaretus* genome at ~1 Mbp. When mapped to the Amur Whitefish *C. ussuriensis* genome, 421 panel loci (85.2%) had a MAPQ score ≥ 30 to regions in 39 out of 40 chromosomes and two scaffolds. Similarly to European Whitefish, no matches occurred in chromosome 40, which is also ~1 Mbp in *C. ussuriensis*. Comparatively, while 464 panel loci (93.9%) successfully amplified in Great Lakes Lake Whitefish *C. clupeaformis* samples, only 430 loci (87.0%) mapped with a MAPQ score of ≥ 30 across 39 of 40 chromosomes (no matches in chromosome 38 of the *C. clupeaformis* genome, ~11 Mbp) and 11 scaffolds suggesting mapping cisco‐based amplicons could underestimate cross amplification potential.

### 3.3 GT‐Seq Genotyping and Genetic Identification of Early Life Coregonines in Lake Superior

A total of 60 early life coregonines were removed for outlying heterozygosity (*n* = 55) and/or contamination scores above our threshold of > 0.50 (*n* = 12), with seven individuals exhibiting both. All but one of the individuals exhibiting high heterozygosity were larvae collected in 2019, and 33 of these were associated with five collection batches that appear to have been cross contaminated at the time of either collection or subsampling for extraction. An additional 18 individuals were dropped from further analysis for missing data above our filtering thresholds for a total of 3066 genotyped early life coregonines that could undergo genetic identification.

Adult references used for assignments exhibited strong clustering by species. For example, the average Q‐scores for references to their associated species cluster in the first of 34 subsetted STRUCTURE runs were *C. artedi* = 0.90, *C. hoyi* = 0.92 and *C. kiyi* = 0.95 (STRUCTURE plot in File S1, Figure S6). Clear differentiation between reference ciscoes can also be seen when the dataset is reduced to the whitefish whitelist (File S1, Figure S5). For all collections of larvae and age‐1 s, more than 90% of samples were able to be identified with the GT‐seq panel (91.7%–100%, Table 3). Of the larvae collected in 2019, most were *C. artedi* (*n* = 569), followed by *C. kiyi* (*n* = 179) and *C. hoyi* (*n* = 5). Genetic species identifications of larvae collected in 2021 showed a similar proportion ( *C. artedi* , *n* = 1522; *C. kiyi* , *n* = 130; *C. hoyi* , *n* = 16). Putative hybrids, those individuals which generated a Q‐score below our 0.70 threshold to assign to a cluster, comprised 11.5% and 9.7% of genotyped larvae in 2019 and 2021, respectively. In the age‐1 collections from 2019 to 2021, most were *C. hoyi* (*n* = 235) followed by a similar number of *C. artedi* (*n* = 39) and *C. kiyi* (*n* = 31). Putative hybrids comprised 4.2%–16.0% of genotyped age‐1 s. Additionally, the whitefish whitelist identified 13 Lake Whitefish in the larval collections and 2 Pygmy Whitefish in the 2020 age‐1 collection. A summary of identified larvae and age‐1 s is presented in Table 3, and *assignPOP* assignment scores and STRUCTURE Q‐scores (as well as dropped individuals) are reported in File S6.

**TABLE 3:** Genetic identifications of early life coregonines collected in Lake Superior 2019–2021. Proportion of putative hybrids (i.e., fishes whose scores were below our assignment threshold) in each collection is listed as a percentage of genotyped individuals.

Species	2019		2020	2021	
	Larvae	Age‐1s	Age‐1s	Larvae	Age‐1s
*C. artedi*	569	4	0	1522	35
*C. kiyi*	179	17	3	130	11
*C. hoyi*	5	17	64	16	154
*C. clupeaformis*	10	0	0	3	0
*P. coulterii*	0	0	2	0	0
Putative hybrid	99 (11.4%)	6 (13.6%)	3 (4.2%)	179 (9.7%)	38 (16.0%)

### 3.4 Preliminary Analysis of Early Life Coregonines in Lake Superior

**FIGURE 3:**
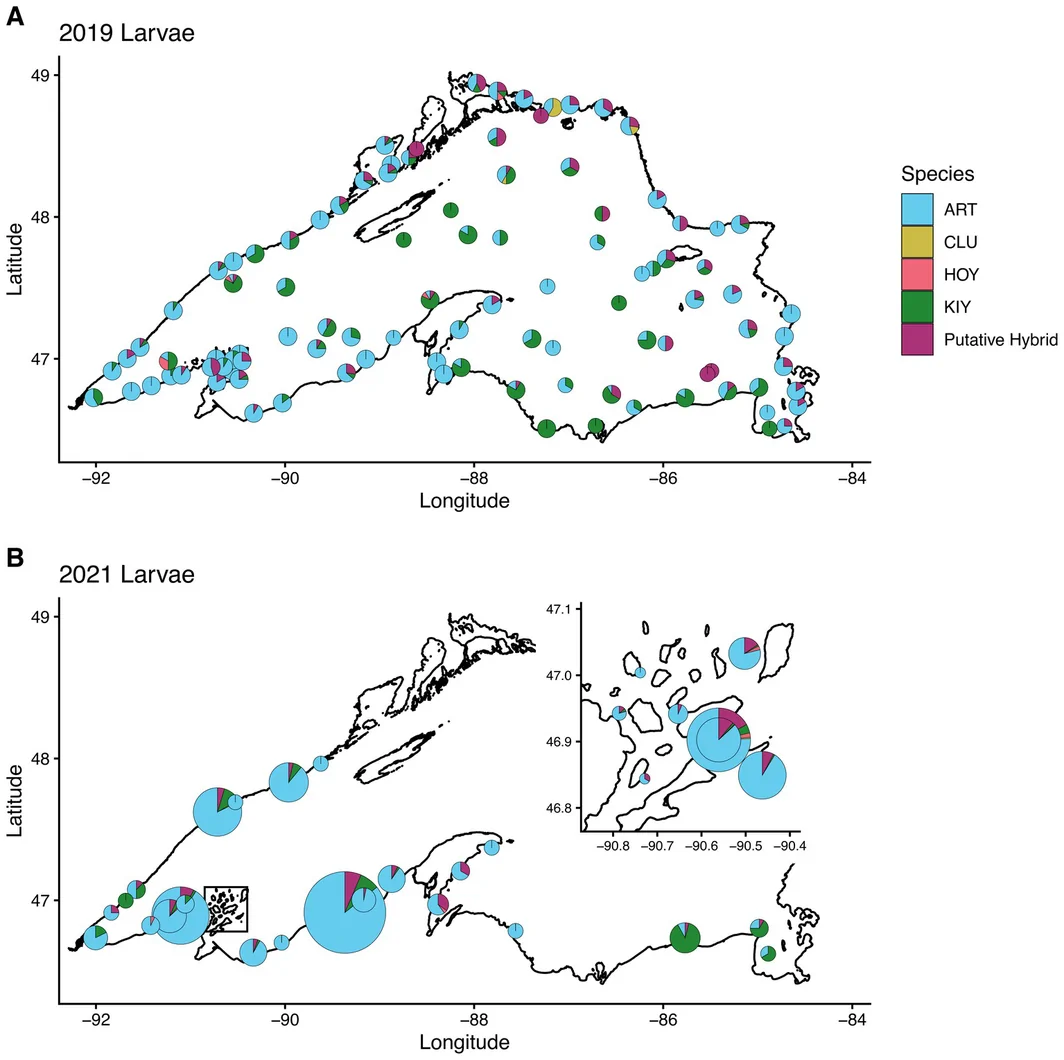
Spatial distribution of 2712 genetically identified larvae collected from Lake Superior in 2019 (A) and 2021 (B). The extent of 2021 larval collections was limited due to travel restrictions during the COVID pandemic. Collections from this year occurred largely in U.S. waters in the region near the Apostle Islands (inset, panel B). Pie chart size is proximal to number of larvae from each collection site. Abbreviations: ART— *C. artedi* (Cisco), CLU— *C. clupeaformis* (Lake Whitefish), HOY— *C. hoyi* (Bloater) and KIY— *C. kiyi* (Kiyi).

Investigation of the spatial distribution of identified coregonines indicated that *C. artedi* and *C. kiyi* are broadly distributed in the surface waters throughout Lake Superior in spring and early summer (Figure 3). The small number of *C. hoyi* larvae collected were in the nearshore areas of the western arm of the lake. The 10 Lake Whitefish larvae caught in 2019 were collected mid‐June to mid‐July nearshore along the northern coastline of Lake Superior, and the three Lake Whitefish larvae caught in 2021 were collected May 20 and 21 nearshore in the western arm of the lake. Only five *C. hoyi* larvae were collected in 2019, so broad comparisons of larval size by collection date were limited to *C. artedi* and *C. kiyi* .

**FIGURE 4:**
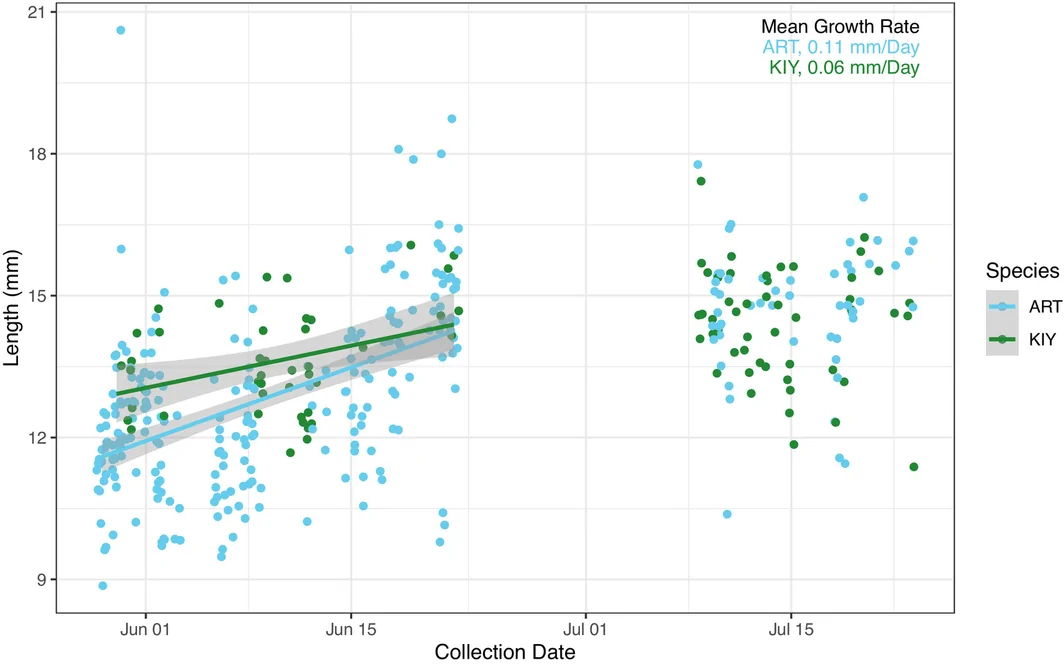
Estimated growth rates of 569 *C. artedi* (ART) and 179 *C. kiyi* (KIY) larvae from late May to July 2019.

The median total length (TL) of *C. artedi* and *C. kiyi* larvae in 2019 was 12.97 mm (range: 8.87–21.04 mm) and 14.19 mm (range: 10.19–17.42 mm), respectively. Of the smaller catches, the median TL of the five *C. hoyi* larvae was 14.08 mm (range: 12.42–14.88 mm), and the median TL of the 10 Lake Whitefish larvae was 16.04 mm (range: 13.42–21.23 mm). Regression analysis indicated an estimated growth rate of 0.11 mm/day for *C. artedi* and 0.06 mm/day for *C. kiyi* from the period of late May to late June 2019 (Figure 4). The median TL of *C. artedi* and *C. kiyi* larvae in 2021 was 12 mm (range: 8–20 mm) and 13 mm (range: 11–20 mm), respectively. Of the smaller catches in 2021, the median TL of the 16 *C. hoyi* larvae was 10 mm (range: 9–12 mm), and the median TL of the three Lake Whitefish larvae was 16.04 mm (range: 13.42–21.23).

**FIGURE 5:**
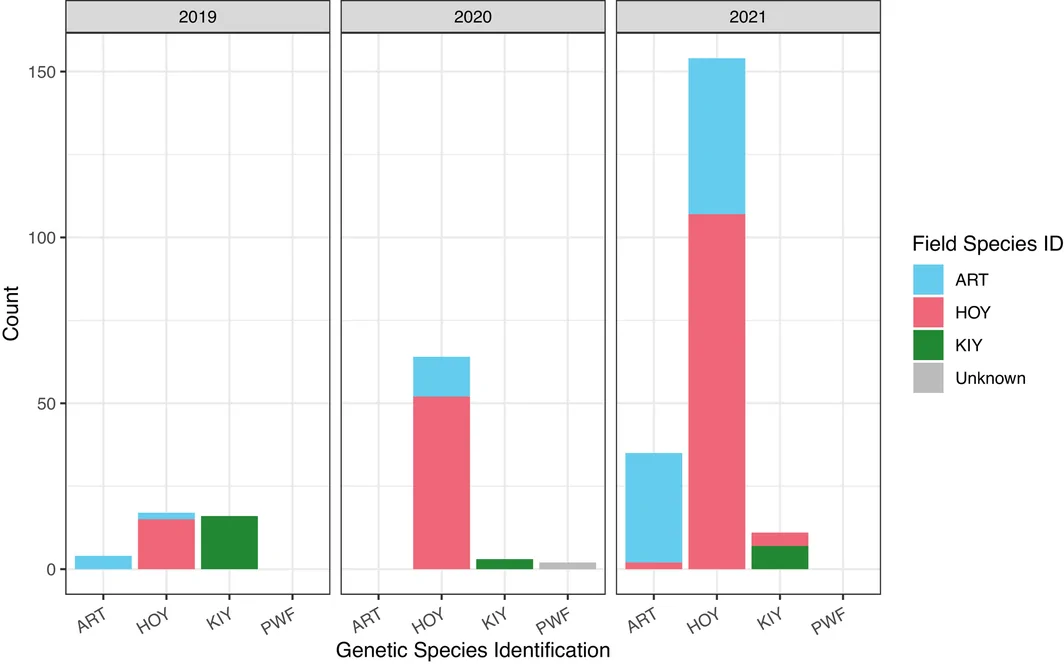
Comparisons of field versus genetic species identifications from 2019 to 2021 age‐1 collections. Abbreviations: ART— *C. artedi* (Cisco), HOY— *C. hoyi* (Bloater), KIY— *C. kiyi* (Kiyi), PWF— *P. coulterii* (Pygmy Whitefish).

Comparisons of field and genetic identifications of age‐1 s highlight the challenges of identifying early life coregonines (Figure 5). Of the three major species, *C. kiyi* was most likely to be correctly identified in the field. All age‐1 s identified as *C. kiyi* genotyped as *C. kiyi* . The 2 Pygmy Whitefishes collected with the 2020 age‐1 s were caught in the Apostle Islands and originally identified as ‘unknown coregonines’ as their total length (25 mm, 26 mm) was significantly smaller than the average for age‐1 ciscoes (in 2020, 137.6 mm). Evaluating age‐1 species identification by collection location indicated that the distribution of juvenile *C. kiyi* is largely offshore and the distribution of juvenile *C. artedi* and *C. hoyi* is more nearshore (File S1, Figure S6).

## 4 Discussion

### 4.1 Development of a GT‐Seq Panel for Cryptic Species Identification

We built a flexible GT‐seq panel of 494 loci containing 1242 target SNPs from RAD‐ and RNA‐seq data capable of identifying distinct members of a *Coregonus* species complex that arose through an evolutionarily recent radiation in North America as well as congener and confamilial coregonines. Our approach to developing a cost‐effective GT‐seq panel for cryptic species identification could be applied to other species and other questions related to species‐ and stage‐specific life history.

The presence of cryptic diversity at any life stage can lead to critical gaps in our understanding of ecological and evolutionary processes with potentially adverse effects on ecosystem and species management (Struck et al. 2018; Hending 2025). When adult stages are cryptic, fundamental demographic parameters such as population size or species range can be significantly overestimated (Schönrogge et al. 2002; Katzner et al. 2011; Clarke et al. 2025). Similarly, unaccounted for cryptic diversity can bias measures of population structure and gene flow. Sheets et al. (2018) compared measures of genetic differentiation when individuals from a cryptic coral complex were correctly identified via a SNP panel versus when they were treated as a single species. Inaccurate characterization of cryptic species led to higher *F*_ST_ and lower estimates of gene flow at local spatial scales and lower *F*_ST_ and higher estimates of long‐term gene flow at broad spatial scales. When early life stages are cryptic, particularly in species with bipartite or complex life histories punctuated by distinct morphology, habitat, or trophic position, species‐specific differences at a pivotal point in life remain unknown. The environment and ecology of early life play a vital role in determining population and species outcomes, including recruitment (Stige et al. 2019), size structure (Laurel et al. 2026), population stability (Knoerr et al. 2022), and local adaptation (Postma and Ågren 2016), underscoring the importance of accessible tools to enable species‐specific investigation.

In the Great Lakes, this GT‐seq panel represents a significant improvement over the two prior methods used to genetically identify cryptic early life coregonines. In Lake Ontario, the cytochrome oxidase subunit I (COI) gene was used to identify 726 Cisco *Coregonus artedi* and Lake Whitefish *C. clupeaformis* larvae collected in 2014 (George et al. 2018). While variation in COI and other mitochondrial regions can distinguish between ciscoes and whitefishes, it cannot distinguish ciscoes from each other (Bernatchez et al. 1991; Turgeon and Bernatchez 2003; Lachance et al. 2021), which is a common challenge for evolutionarily young species (van Velzen et al. 2012). This method was a viable option in Lake Ontario at the time because a century of anthropogenic impacts had reduced the diversity of ciscoes from four species to one (Eshenroder et al. 2016). However, a bi‐national restoration initiative has been underway since 2012 to reintroduce Bloater *C. hoyi* to Lake Ontario (Ontario Ministry of Natural Resources and Forestry 2015; Weidel et al. 2022), and as this effort progresses, COI may no longer be an accurate method for early life stage identification. Additionally, a 2018 study examining emergence and species composition of larval coregonines in the Apostle Islands of Lake Superior used COI to differentiate Lake Whitefish from ciscoes, then genetically identified 193 larvae that barcoded as ciscoes with RAD‐seq (Lachance et al. 2021). Reduced representation sequencing methods such as RAD‐seq can be extremely effective at identifying cryptic diversity (Pante et al. 2015; Herrera and Shank 2016), but can cost up to 5× more per sample (Meek and Larson 2019), significantly limiting the scope of study. Here, we demonstrate the relative ease with which thousands of larvae can be identified with a single, cost‐effective method, enabling research and monitoring of early life stages that often rely on extensive spatial or temporal sampling to achieve robust results (Clark et al. 1999).

### 4.2 Cross Amplification in Congeneric and Confamilial Species

While originally designed to distinguish members of the cisco species complex, we were able to adapt SNP probes for genetic identification of Great Lakes whitefishes with relative ease and high accuracy. Cross amplification was more successful between species within genus than between genera within family, echoing broad patterns observed in microsatellite cross amplification in fishes and other large taxonomic groups such as monocots, arthropods and birds (Barbará et al. 2007). Despite the reduction in success across genera, cross amplification was widespread across the Cisco *C. artedi* genome in both congeneric and confamilial coregonines. Genomic resources for coregonines are still emergent—only two North American species (Cisco *C. artedi* and Lake Whitefish *C. clupeaformis* ) have published genomes (Backenstose et al. 2024; Mérot et al. 2023). Synteny between their chromosomes has not been evaluated in the published literature; however, the high proportion of loci that cross‐amplify in Lake Whitefish across the 38 resolved chromosomes of the *C. artedi* genome and good quality amplicon mapping in 39 out of 40 Lake Whitefish chromosomes indicates these loci remain widespread across the *C. clupeaformis* genome. High synteny is present between Lake Whitefish and the European Whitefish *C. lavaretus* sp. Balchen genomes (Mérot et al. 2023) and similarly between *C. lavaretus* sp. Balchen and the Amur Whitefish *C. ussuriensis* in Asia (Huang et al. 2024). Relatively comparable proportions of panel amplicons mapping with good quality to the genomes of these species and Lake Whitefish indicates synteny could be used as a preliminary predictor for cross amplification success. The only chromosome that panel amplicons did not map to in European and Amur Whitefish is a small, ~1 Mbp ‘super‐scaffold’ (Chromosome 40; De‐Kayne et al. 2020, Huang et al. 2024) detected in both species that De‐Kayne et al. (2020) hypothesized could be part of another chromosome. Our results suggest that panel loci may remain broadly representative of neutral or adaptive genomic variation in these species and could be successfully mined for off target use in other *Coregonus* spp. across the northern hemisphere.

Cross amplification was lower in the *Prosopium* whitefishes, though the smaller number of loci that amplified in these fishes remained widespread across the *C. artedi* genome. Despite the decrease in loci, we were still able to achieve high accuracy in genetic species identification for both Round Whitefish *P. cylindraceum* and Pygmy Whitefish *P. coulterii* from the Great Lakes. No genomes have been published for any *Prosopium* spp. so inferences from synteny cannot be made as to whether cross‐amplifying loci may be widespread across independent *Prosopium* chromosomes. Karyotypes for *P. cylindraceum* and *P. coulterii* indicate nearly the same number of chromosomes (2*n* = 78 and 82, respectively; Booke 1968) as in ciscoes (2*n* = 80; Booke 1968; Phillips et al. 1996); however, *P. cylindraceum* and *P. coulterii* are the most ancestral of the genus (Vuorinen et al. 1998), and karyotypes of the four other *Prosopium* spp. show successively reduced numbers of chromosomes (2*n* = 64–74; Booke 1970, 1974) from Robertsonian translocations facilitating the process of rediploidization and speciation (Booke 1974; Phillips and Ráb 2001). Whether the approximately 200 panel loci that cross‐amplified in *P. cylindraceum* and *P. coulterii* would continue to successfully amplify across the breadth of *Prosopium* spp. in the face of significant chromosomal rearrangements remains to be tested.

### 4.3 Early Life Coregonines in Lake Superior

The distributions of early life coregonines appear heavily influenced by mobility and species‐associated emergence timing. In Lake Superior, pelagic *C. artedi* spawn in nearshore waters of depths between 15 and 30 m (Koelz 1929; Yule et al. 2006), while deepwater *C. kiyi* spawn offshore in depths > 100 m (Vinson et al. 2023). Larvae of both species were consistently collected in the upper metre of the lake in both near and offshore tows, suggesting larval orientation is largely driven by yolk‐sac buoyancy, positive phototaxis and passive dispersal via currents. Conversely, age‐1 s of these species segregated to nearshore midwater ( *C. artedi* ) and offshore deepwater ( *C. kiyi* ) following the known species‐specific distributions of juveniles and adults of these species (Stockwell et al. 2006; Yule et al. 2013; Rosinski et al. 2020). The fact that *C. hoyi* was rarely collected in larval tows from May to July likely reflects spawn and emergence timing. The three species spawn in consecutive months in the fall and winter, beginning with *C. artedi* (November, Koelz 1929), followed by *C. kiyi* (late December‐late January; Vinson et al. 2023), and then *C. hoyi* (February–March, Dryer and Beil 1968). Continued sampling into August and September or at greater water column depths could better characterize the distribution of newly emerged *C. hoyi* larvae to confirm if their distributional patterns mimic that seen in *C. artedi* and *C. kiyi* .

Growth rates and larval size indicated species‐specific differences between *C. artedi* and *C. kiyi* larvae. Notably, *C. artedi* larvae were consistently caught at smaller sizes than *C. kiyi* larvae in both 2019 and 2021. Whether this is reflective of a smaller size at hatch for *C. artedi* , growth that may occur between hatch in deeper water and presence in surface waters of *C. kiyi* , or some other physiological difference is unknown. Hatchery collections of *C. artedi* have been established for stocking purposes since the late 1800s (McKenna et al. 2025) and could be used to more comprehensively examine larval development. However, the rearing of *C. kiyi* eggs has only been documented once (Todd et al. 1981) as the winter spawning period and depth of *C. kiyi* makes collection of spawning individuals notoriously difficult. Vinson et al. (2023) were able to collect spawning *C. kiyi* from Lake Superior in 2017 to 2019 and determined that average egg diameter in *C. kiyi* (~1.7 mm) was not significantly different than *C. artedi* (Koenigbauer et al. 2022). Despite the presence of smaller *C. artedi* larvae in surface collections, higher growth rates were observed in *C. artedi* than in *C. kiyi* , though the rates presented here should be viewed cautiously. Adult *C. artedi* is substantially larger than adult *C. kiyi* (Koelz 1929) so a faster growth rate would not be unexpected; however, more robust estimates than those presented here could be obtained in the future by better controlling for geographic region across time and potential gear bias on larval size.

The presence of putative hybrids in larval and age‐1 s samples reflects evidence for hybridization previously seen in coregonines across the northern hemisphere. Hybridization and speciation reversal driven by anthropogenic impacts like eutrophication and non‐native species introduction has been documented among *Coregonus* spp. in number of European Alpine and subarctic lakes (Kahilainen et al. 2011; Vonlanthen et al. 2012; Frei et al. 2023) and arctic lakes (Reist et al. 1992). Hybridization has also been seen between species pairs of Lake Whitefish *C. clupeaformis* in Canadian lakes and has been hypothesized to be associated with incipient ecological speciation (Lu et al. 2001; Rougeux et al. 2017). Recently with both RAD‐seq and whole‐genome sequencing data, wild captured hybrids were documented in Lake Superior (Ackiss et al. 2020; Lachance et al. 2021; Backenstose 2024). Unlike many of the Alpine and subarctic lake populations, the nature of hybridization among ciscoes in Lake Superior is unstudied. The average proportion of putative larval and age‐1 s hybrids detected in our study (~11%) was similar to the hybrid rate previously estimated among adults (~9%; Ackiss et al. 2020) but was nearly triple the proportion of hybrid larvae estimated by Lachance et al. (~4%; 2021). The increase in putative hybridization rates in larvae detected here may be the result of a more than seven‐fold increase in sample numbers, a reflection of stochastic differences between year classes, or evidence of ecological change breaking down pre‐zygotic isolating mechanisms. Hybrid crosses among North American coregonines made in experimental settings are not well documented, but the few studies performed to date have found that while fertilization is successful, most embryos die before or shortly after hatch (Moenkhaus 1910; Garside and Christie 1962). Long term monitoring in Lake Superior of both larvae and adults could illuminate whether the proportions of putative hybrids are indicative of natural variation in an evolutionarily recent ecological species radiation that is undergoing speciation‐with‐gene flow or evidence that ecosystem change may be driving speciation reversal.

Comparisons of field and genetic identifications of age‐1 s reflected the challenges that cryptic early life stages can present to accuracy in identifications. Misidentifications of age‐1 *C. kiyi* were relatively rare, with only a few individuals from 2021 originally identified as *C. hoyi* . Misidentifications of this type are not uncommon in adults as well. Early descriptions of the species complex noted *C. hoyi* and *C. kiyi* could be confused with each other in Lake Superior (Koelz 1929), and genetic analysis of adults in the Apostle Islands found three out of 24 individuals originally identified as *C. hoyi* genotyped as *C. kiyi* (Ackiss et al. 2020). In contrast, no misidentifications between adult *C. artedi* and *C. hoyi* were seen by Ackiss et al. (2020), and these were the most common misidentifications in age‐1 s here. This may be an indication of variance between individuals performing identification or reduced accuracy or consistency in the characters used to distinguish the two in early life. With new accessibility to a tool for genetically confirming species identification, characters for morphological identification of age‐1 s can be reevaluated to increase accuracy.

These preliminary results illustrate the value of a simple, targeted and efficient solution for cryptic early life species identification, particularly when species‐specific outcomes can be variable. In the Great Lakes, the deepwater species of the *C. artedi* species complex were inordinately impacted by the rapid ecological changes that have occurred over the past century (Eshenroder et al. 2016). More recently, however, declines in the shallow water coregonine Lake Whitefish *C. clupeaformis* have been observed (Ebener et al. 2021; Cunningham and Dunlop 2023), while some shallow water *C. artedi* populations appear to be stable or increasing (Tingley III et al. 2025). Early life in fishes is a critical period underpinned by rapid changes in morphology, ecology and habitat use and high mortality (Chambers and Trippel 2012). Latent or carry‐over effects—delayed effects of early life environment and experience—have also been shown to impact outcomes in fishes ranging from juvenile survival (Gagliano et al. 2007) to sex‐determination (Shima et al. 2024). A better understanding of the early life period in coregonines could play a crucial role in determining the nature of differential responses to an ecosystem undergoing rapid environmental and ecological change. This panel now provides a cost‐effective method for future research of the *C. artedi* species complex at the species‐specific level across the whole life history spectrum, opening the door to new insights into the mechanisms by which species‐specific adaptation has driven speciation and outcomes in coregonines and demonstrating a flexible method that could be applied to other taxa that exhibit cryptic species or life stages.

## Author Contributions

**A.S.A.** – RAD‐seq library preparation and genotyping, GT‐seq panel development/optimization, GT‐seq genotyping, data analysis, lead manuscript development/writing; **M.R.V.** – project conception, collected samples, manuscript writing/editing; **A.J.R.** – performed sequencing, data analysis, data archiving, manuscript writing/editing; **K.M.G.** – supported GT‐seq panel development/optimization, manuscript editing; **T.J.K.** – provided transcriptomic data, manuscript writing/editing; **J.V.S.** – performed DNA extraction, GT‐seq library preparation, data analysis; **W.S.** – project conception, collected samples, manuscript editing; **D.L.Y.** – collected samples, manuscript writing/editing; **W.A.L.** – project conception, manuscript editing. All authors approved the final draft submitted for publication.

## Funding

This research was funded by the U.S. Environmental Protection Agency through a Great Lakes Research Initiative grant.

## Disclosure

Benefit‐Sharing: This work was enabled by existing and newly developed research collaborations with scientists and fishery managers from both the United States and Canada providing genetic samples. Contributors to this research include international, federal, state, tribal, First Nation and provincial agencies and scientists which are described in the Methods, Acknowledgements and Sample Metadata (see above). The results of this research have been shared with the provider communities and the broader scientific community. This research addresses a bi‐national priority concern, in this case the conservation of critical prey fishes in the Laurentian Great Lakes of North America. Lastly, as described above, all data have been shared with the broader public via appropriate biological databases.

## Ethics Statement

This study did not involve experimentation on live animals. This research used fishes of a variety of life stages that were dead upon collection. Permitted collection was performed by several agencies, including the Great Lakes Fishery Commission, Ontario Ministry of Natural Resources, Chippewa Ottawa Research Authority, Sault Ste. Marie Tribe, Little Traverse Bay Band of Odawa Indians, Wisconsin Department of Natural Resources, United States Fish and Wildlife Service and the United States Geological Survey. Our work follows all ethics and research integrity standards.

## Conflicts of Interest

The authors declare no conflicts of interest.

## Supporting information


**Table S1 (File S1):** The eight RADseq locus combinations used for simulating panel performance. Combinations targeted the top loci with the highest differentiation (*G'*_ST_) or diversity (*H*_e_) estimates with both SNP and haplotype datasets. Differentiation and diversity estimates were generated for two datasets: (1) Lake Superior individuals, representing the three major extant species and (2) all individuals (basin‐wide). The locus combination in **bold** was chosen for panel development.
**Figure S1 (File S1):** Self‐assignment proportions of reporting groups. Panel A: Preliminary assignment tests indicated that two groups—Grand Portage (A‐SGP) and the North Channel/Georgian Bay region (A‐HNC) – had more than 25% of individuals assign to other locations (red bars). Panel B: Final reporting groups used to assess candidate panels, with individuals from A‐SGP and A‐HNC combined with closely associated groups (A‐SUP and A‐HBI‐SM‐NC, respectively).
**Figure S2 (File S1):** Simulated performance of different combinations of loci for assignment of individuals to reporting units. Reporting units and locus combinations are described in Table 1 in the manuscript & and Table S1.
**Figure S3 (File S1):** Round Whitefish, Pygmy Whitefish and Lake Whitefish BAM alignments in IGV for GT‐seq panel locus 29,088 (A) and 57,022 (B). Panel A: the first probe (blue lines in bottom panel of window) for locus 29,088 was redesigned to account for the variant SNP in Round Whitefish. Panel B: probes were not redesigned for locus 57,022 because of variant complexity (multiple fixed SNPs in Round and Pygmy Whitefishes that would result in two or more degenerate sites in each probe).
**Figure S4 (File S1):** Assignment accuracy plot for Lake Whitefish (CLU), Round Whitefish (PRO) and Pygmy Whitefish (PWF) using a whitelist of 428 SNPs that were polymorphic in *Prosopium* whitefishes. All combinations of loci and individuals tested resulted in 100% assignment accuracy.
**Figure S5 (File S1):** Three‐dimensional principal components analysis of reference individuals using a whitelist of 428 SNPs that were polymorphic in *Prosopium* whitefishes. lmCLUrefs—Lake Michigan Lake Whitefish, lsCLUrefs—Lake Superior Lake Whitefish, lsARTrefs— *C. artedi* , lsHOYrefs— *C. hoyi* , lsKIYrefs— *C. kiyi* , PROrefs—Round Whitefish, PWFrefs—Pygmy Whitefish.
**Figure S6 (File S1):** Spatial distribution of genetically identified age‐1 s collected from Lake Superior in 2019 to 2021. Pie chart size is proximal to number of larvae from each collection site.

**File S2:** GT‐seq panel library preparation protocol.

**File S3:** GT‐seq panel probe file for genotyping with GTscore.

**File S4:** GT‐seq panel primers (forward _F, reverse _R), with the small RNA and read 2 sequencing primer sequences used. Primers that cross amplified in whitefishes are indicated in Columns (C–E).

**File S5:** Whitelist of 428 Loci for Identification of Whitefishes.

**File S6:** Collection information, AssignPOP and STRUCTURE Q‐scores, and Genetic ID for early life coregonines colleced from 2019 to 2021 in Lake Superior. More detailed collection information is available on ScienceBase, https://doi.org/10.5066/P13W68YB. Any use of trade, firm, or product names is for descriptive purposes only and does not imply endorsement by the U.S. Government.

## Data Availability

RAD sequence data from *C. artedi* , *C. hoyi* and *C. kiyi* from the Apostle Islands and *C. artedi* from Boot Island were previously archived on NBCI sequence read archive (Project IDs: PRJNA600818, PRJNA1266121). RNA‐seq reads from *C. artedi* , *C. hoyi* and *C. kiyi* from Lake Superior were previously archived on NCBI sequence read archive (Project ID PRJNA659559). Sample and sequence archive data for all samples genotyped with RAD‐seq and GT‐seq can be found on ScienceBase (https://doi.org/10.5066/P13W68YB, https://doi.org/10.5066/P145DC3U).
